# Adherence and Metabolic Outcomes of Early and Late Time-Restricted Eating with Energy Restriction vs. Energy Restriction Alone: A 6-Month Follow-Up

**DOI:** 10.3390/nu18061004

**Published:** 2026-03-21

**Authors:** Tanja Črešnovar, Bernarda Habe, Nina Mohorko, Saša Kenig, Zala Jenko Pražnikar, Ana Petelin

**Affiliations:** Faculty of Health Science, University of Primorska, Polje 42, 6310 Izola, Slovenia; tanja.cresnovar@upr.si (T.Č.); bernarda.habe@upr.si (B.H.); nina.mohorko@upr.si (N.M.); sasa.kenig@upr.si (S.K.); zala.praznikar@upr.si (Z.J.P.)

**Keywords:** early time-restricted eating, late time-restricted eating, energy restriction, overweight, obesity

## Abstract

**Background/Objectives:** Long-term weight maintenance remains challenging with conventional dietary strategies due to various barriers. Time-restricted eating (TRE) has recently attracted attention as a potential approach to improve adherence, but evidence on long-term maintenance is limited. We investigated the 6-month follow-up (6FU) of early time-restricted eating with energy restriction (eTRE + ER), late time-restricted eating with energy restriction (lTRE + ER) and energy restriction alone (ER). **Methods:** This 6FU included 69 of 93 participants from a previously conducted 3-month intervention (3INT). After the intervention, participants returned to free-living conditions without dietary guidance. Outcomes included adherence, perceived barriers, body composition, blood pressure, cardiometabolic risk factors, metabolic hormones, subjective appetite, and dietary intake. **Results:** Adherence of at least ≥5 days per week was low: 7.7% (eTRE + ER), 18.2% (lTRE + ER), and 9.5% (ER). Reduced adherence during the 6FU was associated with a partial reversal of improvements in body mass, body composition, cardiometabolic risk factors, metabolic hormones, and subjective appetite observed during the 3INT. Analysis of perceived barriers showed that environmental and psychosocial barriers were significant predictors of changes in body mass during the 6FU, while environmental and behavioral barriers were associated with extension of the eating window. These associations were most pronounced in the eTRE + ER group. **Conclusions**: During the 6FU, differences between dietary strategies gradually diminished, although some remained clinically meaningful. Long-term adherence was low across all three dietary strategies, with psychosocial, environmental, and behavioral barriers particularly evident in the eTRE + ER group. Further research is needed to confirm long-term adherence before TRE + ER interventions can be widely applied in clinical practice.

## 1. Introduction

Obesity is a major public health concern with a rising prevalence worldwide [1,2]. While many dietary strategies promote weight loss, few achieve lasting improvements in population health [3]. Conventional daily energy restriction (ER) of 25–30% typically results in a 5–9% weight loss within 3–6 months but is difficult to sustain in the long term [3,4,5,6,7,8]. Weight regain is influenced by biological, behavioral, psychosocial and environmental factors [9], but long-term adherence remains the strongest predictor of success [5]. Therefore, developing dietary strategies that enhance adherence and the durability of outcomes is a key priority [5,10].

One emerging approach is time-restricted eating (TRE), which confines food intake to a daily window of 10 h or less. TRE may promote weight loss and cardiometabolic health by aligning food intake with circadian rhythms and metabolic processes [11,12,13,14]. Its simplicity—focusing on when to eat rather than what to eat—may also support greater adherence over time [5]. Additionally, short-term studies have shown that individuals following TRE often spontaneously reduce their daily energy intake by approximately 500 kcal per day [12,15]. However, existing human studies on TRE are of relatively short duration. The results are limited to the intervention period, with a lack of follow-up assessments [5]. Consequently, the long-term maintenance of this dietary strategy and the potential barriers to its sustainability remain unclear.

To address these gaps, we conducted a 6-month follow-up study (6FU) after completion of a 3-month intervention (3INT) comprising early time-restricted eating with energy restriction (eTRE + ER), late time-restricted eating with energy restriction (lTRE + ER), and ER alone, with the aim of evaluating the maintenance of the intervention under free-living conditions and identifying potential barriers to adherence. In addition, we examined changes in body mass, body composition, metabolic risk factors, metabolic hormones and subjective appetite from the end of the 3INT to the end of the 6FU.

## 2. Materials and Methods

### 2.1. Study Design

This study presents a 6FU analysis after a completed 3INT clinical trial, carried out at the University of Primorska Faculty of Health Sciences between March and June 2023. The 6FU took place between June and December 2023, with measurements taken in December 2023. The study was approved by the Slovenian National Medical Ethics Committee (No. 0120–557/2017/4; Ministry of Health, Republic of Slovenia) and registered at ClinicalTrials.gov, identifier NCT05730231. All participants completed an informed consent form.

### 2.2. The 3INT and 6FU

The protocol for the 3INT has been described in detail previously [16]. In summary, participants were recruited via online platforms, social media, and local newspapers and completed an entry questionnaire on demographics, lifestyle, health and anthropometrics. The inclusion criteria were met by adults aged 18 to 60 years with a body mass index between 25 kg/m^2^ and 35 kg/m^2^ who met at least one component of the metabolic syndrome, based on established criteria [17]. Exclusion criteria included taking medication for hypertension or hypercholesterolemia, daily eating window of less than 11 h, pregnancy or breastfeeding, smoking, participation in a weight loss program, presence of chronic diseases (e.g., cardiovascular, gastrointestinal, oncological, hematological diseases, etc.), a diagnosed eating disorder or history of an eating disorder, shift work, alcohol consumption greater than 2 servings per day for men and greater than 1 serving per day for women, or use of dietary supplements that could influence the main results of the study. Participants were allocated to three intervention groups (eTRE + ER, lTRE + ER, or ER) based on their Morningness-Eveningness Questionnaire (MEQ) score [18], as previously described [16]. Participants with a total MEQ score >58 were classified as extreme morning, morning, or intermediate types and randomly assigned in a 2:1 ratio to either the eTRE + ER or ER group. Similarly, participants with a MEQ score ≤58 were classified as extreme evening, evening, or intermediate types and randomly assigned to either the lTRE + ER or ER group, again in a 2:1 ratio. The variables used for stratified randomization between the two groups were sex, age (18–39 and 40–60 years), and BMI (25.0–29.9 kg/m^2^ and 30.0–34.9 kg/m^2^). Randomization with stratification was performed using the free open-source desktop application MinimPy (https://sourceforge.net/projects/minimpy) (accessed on 25 February 2023) [19]. After randomization, the median MEQ score was 64 in the eTRE + ER group, 54 in the lTRE + ER group, and 58 in the ER group.

**Figure 1:**
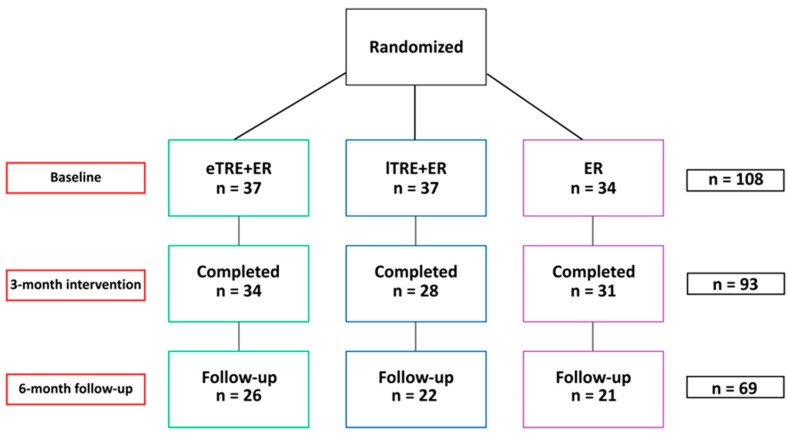
Flow diagram of participants. Abbreviations: ER, energy restriction; eTRE, early time-restricted eating; lTRE, late time-restricted eating; n, number of participants.

The 3INT included four measurements and three motivational sessions. All participants received a dietary plan of three meals per day, with a standardized macronutrient distribution of 45–50% carbohydrates, 15–20% protein and 30–35% fat, and a prescribed daily energy reduction of 2100 kJ (−500 kcal) per day, calculated based on individual resting metabolic rate and self-reported physical activity level. During fasting periods, participants were allowed to consume only plain water and unsweetened tea. They also attended monthly motivational meetings with a dietitian, which provided nutrition education, including guidance on meal composition, energy and nutrient values of food, food preparation and emotional eating. Adherence was monitored through a daily dietary log, in which participants recorded compliance with the prescribed TRE (with a ± 30 min tolerance per day) and the prescribed ER. Participants were excluded from the intervention if they failed to comply with the study protocol for four consecutive days. Moreover, at baseline and after two months of the intervention, participants completed a 3-day food record. Of the 108 enrolled, 93 completed the intervention (eTRE + ER: n = 34; lTRE + ER: n = 28; ER: n = 31) (Figure 1).

After the 3INT, participants returned to free-living conditions without dietary guidance. At 9 months, the 6FU study was performed; participants were invited for repeated measurements and to complete a 3-day food diary, an online questionnaire on appetite, and a questionnaire assessing long-term adherence and perceived barriers. Ultimately, 6FU data were obtained from 69 participants (eTRE + ER: n = 26; lTRE + ER: n = 22; ER: n = 21) (Figure 1).

### 2.3. Measurements

Body composition, resting metabolic rate, blood pressure and blood sampling were conducted using the same standardized procedures as in previous assessments [16]. All measurements were performed at baseline, monthly during the 3INT and at the end of the 6FU. All assessments were performed in the morning following a 12 to 15 h overnight fasting. Body composition was assessed using Tanita MC-980MA bioelectrical impedance analyzer (BIA) (Tanita Corporation, Arlington Heights, IL, USA) and dedicated software (GMON Pro-Tanita, version 3). Waist circumference was measured at the mid-axillary line. Blood pressure was measured once after at least 5 min of rest using an automatic device (Tensoval comfort classic). Venous blood samples were collected in 9 mL vacuum test tubes (Greiner Bio-One, Kremsmünster, Austria). Serum was separated by centrifugation at 2000× *g* for 10 min, frozen and stored at −80 °C until analysis. Serum concentrations of biochemical parameters (fasting glucose, lipid profile) were measured with a Cobas c111 analyzer (Roche, Basel, Switzerland). Serum leptin and acylated ghrelin were measured using the ELISA immunoenzymatic assay (BioVendor, Brno, Czech Republic). Assay sensitivity was 0.2 ng/mL for leptin and 4 pg/mL for acylated ghrelin. Inter-assay and intra-assay CVs were typically < 10%.

Participants’ subjective appetite (hunger, fullness, stomach fullness, desire to eat, and capacity to eat) was assessed in the morning at baseline, after the first month, at the end of 3INT, and at the end of 6FU using a visual analog scale (VAS). The VAS consisted of a 10 cm (100 mm) horizontal line anchored at each end with opposing statements (0 = “not at all” and 100 = “extremely”). The line was visually divided into 10 equal units. Participants were instructed to place a mark on the line corresponding to their current subjective feelings. The distance from the left anchor to the mark was measured in millimeters and used for statistical analysis [20].

At the end of the 6FU, participants completed a two-part questionnaire on barriers to long-term adherence. In the first part, participants reported whether they continued to follow the dietary protocol assigned during the 3INT (eTRE + ER, lTRE + ER, or ER) and indicated the average number of days per week they adhered to the prescribed dietary strategy. Response options were categorized as 0 days, 1 day per week, 2 days per week, 3 days per week, 4 days per week or 5 or more days per week. This measure was used to assess self-reported adherence during the free-living follow-up period. In the second part, participants responded to an open-ended questionnaire regarding any barriers they experienced in sustaining the prescribed TRE. Additionally, the eating window during the follow-up period was self-reported using a three-day food diary, in which participants recorded the start and end times of their daily eating window.

### 2.4. Statistical Analysis

Statistical analysis was performed using SPSS version 29.0 (IBM Corp., Armonk, NY, USA). Data are presented as mean ± SD or mean (95% CI). All continuous variables were checked for normality by using the Shapiro–Wilk test. Baseline characteristics were summarized using descriptive statistics. Categorical variables are presented as frequencies and were analyzed using the chi-square test.

Changes in body mass, body composition, cardiometabolic risk factors, metabolic hormones, food intake and subjective appetite were analyzed at two points, from baseline to the end of the 3INT, and from the end of the 3INT to the end of the 6FU. All outcomes were analyzed using one-way ANOVA with Bonferroni post hoc test for normal distribution or Kruskal–Wallis test for non-normal distribution. Within-group comparisons were performed using paired *t*-tests or Wilcoxon signed-rank tests, as appropriate. A significance level was defined as *p* < 0.05 for all statistical tests.

A codebook approach was employed for data analysis regarding barriers to adherence to eTRE + ER, lTRE + ER, and ER using the Atlas.it software. Our analysis was based on four predefined main codes (i.e., biological, behavioral, psychosocial and environmental), which were derived from previous studies identifying barriers to TRE [21,22]. Based on these main codes, subcodes were established to further define key influencing factors. Specifically, for the main code biological barriers, the subcodes were hunger, energy, and physical activity; for behavioral barriers, the subcodes were eating behaviors and 24 h activity behaviors; for psychosocial barriers, the subcodes were self-monitoring and psychological impact; and for environmental barriers, the subcodes were social situations, schedules, and time of the year [21,22].

To identify predictors of changes during the 6FU, multiple linear regression analyses were performed. Dependent variables included changes in body mass, eating window duration, and energy intake, all calculated as the difference between the end of the 3INT and the 6FU. Independent variables comprised perceived adherence barriers, categorized into four predefined domains: environmental, behavioral, psychosocial and biological barriers. Qualitative codes derived from the open-ended responses were transformed into quantitative variables using a simple count approach. Specifically, for each participant, the number of mentions within each predefined category was counted and subsequently used as a numerical predictor in the regression models. Analyses were conducted separately for each group (eTRE + ER, lTRE + ER, ER) and for the pooled sample. Binary logistic regression analyses were used to identify predictors of body mass maintenance during the 6FU. Participants were classified according to whether they maintained body mass (≤+2%) or gained body mass (≥+2%) between the end of the 3INT, and the end of the 6FU in line with previously published criteria [23].

## 3. Results

### 3.1. Baseline Characteristics of the Participants That Responded to the 6FU

The results of the primary and secondary metabolic outcomes for the 93 participants who completed the 3INT study have been reported elsewhere [16]. Of these 93 participants, 6FU data were obtained from 69 (74%) participants, who completed the 6FU assessments. The response rates for the latter were 76.5% (n = 26) in the eTRE + ER, 78.6% (n = 22) in the lTRE + ER group, and 67.7% (n = 21) in the ER group (Figure 1). For the 24 (26%) participants who did not respond to the follow-up invitation, the reasons for non-participation were not obtained.

The baseline characteristics of the participants who responded to the 6FU invitation are shown in Table 1. At baseline, all three groups were comparable in terms of socio-demographic characteristics, body composition, cardiometabolic risk factors, metabolic hormones, food intake, subjective appetite, and daily eating window (all *p*_s_ > 0.05). Statistically significant differences were found in place of residence, baseline diastolic blood pressure, total cholesterol, and LDL cholesterol. Participants in the eTRE + ER group were significantly more likely to be from a village compared to those in the lTRE + ER group and had lower total cholesterol and LDL cholesterol levels than participants in the lTRE + ER group (*p* = 0.026; *p* = 0.025; *p* = 0.033). In addition, participants in the eTRE + ER group had significantly higher diastolic blood pressure than participants in the ER group (*p* = 0.029).

### 3.2. Adherence, Energy Intake, and Eating Window in the 6FU

At the end of the 6FU, participants reported adherence to the dietary protocols prescribed at the beginning of the 3INT (eTRE + ER, lTRE + ER, and ER). Mean adherence during the 6FU was 2.7 ± 1.3 days per week in the eTRE + ER group, 3.1 ± 1.4 days per week in the lTRE + ER group, and 2.6 ± 1.4 days per week in the ER group. Participants who adhered to the prescribed dietary strategy for ≥5 days per week were classified as demonstrating consistent adherence. Adherence at this level was observed in 7.7% of participants in the eTRE + ER group, 18.2% in the lTRE + ER group, and 9.5% in the ER group, with no statistically significant differences between groups. This threshold was selected based on prior intervention studies indicating that adherence to eTRE for ≥5 days per week is sufficient to achieve meaningful improvements in body mass and cardiometabolic health [23].

**Figure 2:**
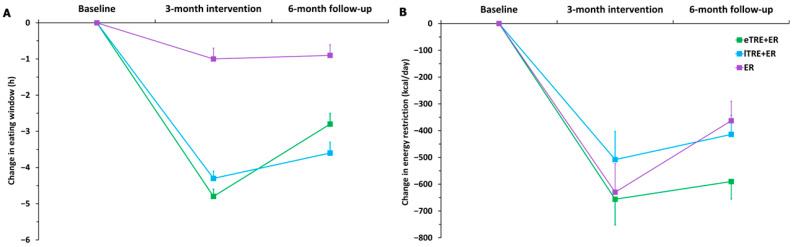
(**A**) Change in eating window at 3INT and 6FU; (**B**) Change in energy intake at 3INT and 6FU. Analyses were performed on 69 participants. Values are presented as mean change with 95% confidence intervals (95% CI).

Table 2 presents changes in eating window, energy restriction and macronutrient distribution in the eTRE + ER, lTRE + ER and ER groups from baseline to the end of the 3INT and from the end of the 3INT to the end of the 6FU. Overall, during the 3INT, all groups showed significant changes with large effect sizes in energy intake and eating window. Reductions in energy intake were observed in all groups (all *p*_s_ < 0.001, Cohen’s *d* ≥ 0.800). However, reduced eating window was significantly more pronounced in the TRE + ER groups than in the ER group (Table 2; Figure 2A,B).

During the 6FU, no significant differences were observed between groups (Table 2; Figure 2B), despite the fact that participants in the ER group significantly increased their energy intake (*p* = 0.003) compared to the end of the 3INT. Participants in both TRE + ER groups significantly extended their eating window compared to the end of the 3INT (eTRE + ER, *p* < 0.001; lTRE + ER, *p* = 0.005), while participants in the ER group maintained the same eating window as in the 3INT. Moreover, the eTRE + ER showed a significantly greater increase in eating window compared to lTRE + ER and ER groups (Figure 2A).

Regarding the distribution of macronutrients, in line with our parent study, participants in all groups increased their percentage of protein intake during the 3INT (eTRE + ER, *p* < 0.001; lTRE + ER, *p* < 0.001; ER, *p* < 0.001). During the 6FU, a significant decrease in the percentage of protein intake was observed in the lTRE + ER and ER groups. In particular, the decrease was significantly more pronounced in the ER group (−4.4 ± 3.1%) than in the eTRE + ER group (−1.0 ± 4.4%) (*p* = 0.004). No significant differences were observed in the proportion of fat or carbohydrate intake during the 3INT and the 6FU (Table 2).

### 3.3. Changes in Body Mass, Body Composition, and Cardiometabolic Risk Factors from Baseline to the End of the 3INT, and from the End of the 3INT to the End of the 6FU

During the 3INT, in 69 participants who attended the follow-up assessments, significant improvements with large effect sizes (all Cohen’s *d* ≥ 0.8) were observed in body mass, BMI, fat mass, waist circumference, fat-free mass, and muscle mass across all three groups (all *p*_s_ < 0.001; Table 3). Between-group differences were observed for BMI between the eTRE + ER and ER groups (*p* = 0.017) and fat mass reduction, which was greater in the eTRE + ER group compared with the lTRE + ER (*p* = 0.003) and ER groups (*p* = 0.021). Moreover, systolic blood pressure significantly decreased within the eTRE + ER group (*p* < 0.001) and the lTRE + ER group (*p* < 0.01). In the ER group, systolic blood pressure also decreased, although this reduction was not statistically significant. No significant differences were observed between groups. In contrast, diastolic blood pressure significantly decreased within all three groups with large effect sizes (all *p*_s_ < 0.001, all Cohen’s *d* ≥ 0.8). A significant difference was observed between the eTRE +ER and ER groups (*p* = 0.006). During the intervention, no statistically significant differences were observed between the groups in fasting glucose, total cholesterol, LDL cholesterol, HDL cholesterol, or triacylglycerols (Table 3), although there was a statistically significant reduction found within the eTRE + ER group in fasting glucose (*p* < 0.01), total cholesterol (*p* < 0.01), and LDL cholesterol (*p* < 0.001).

During the 6FU, the eTRE + ER and ER groups showed a significant increase with small to medium effect sizes in body mass (*p* < 0.01; *p* < 0.01, respectively), BMI (*p* < 0.01; *p* < 0.01, respectively), fat mass (*p* < 0.01; *p* < 0.001, respectively) and systolic blood pressure (*p* < 0.05; *p* < 0.05, respectively). Additionally, diastolic blood pressure also increased significantly with medium effect size in the eTRE + ER group (*p* < 0.01). Regarding biochemical parameters, a significant increase with a small effect size was found in the eTRE + ER group in fasting glucose, total cholesterol, and LDL cholesterol, while HDL cholesterol increased significantly with large effect sizes (all *p*_s_ < 0.001, all Cohen’s *d* ≥ 0.800) within all three groups. No significant differences were observed between the groups in changes in cardiometabolic risk factors (Table 3).

### 3.4. Changes in Metabolic Hormones and Subjective Appetite from Baseline to the End of the 3INT and from the End of the 3INT to the End of the 6FU

Table 4 presents changes in metabolic hormones and subjective appetite from baseline to the end of the 3INT and from the end of the 3INT to the end of the 6FU.

During the 3INT, leptin levels and hunger decreased significantly in all groups (*p*_s_ < 0.001; *p*_s_ < 0.01, respectively). The decrease in leptin was significantly greater in the eTRE + ER group than in the ER group (*p* = 0.001). Stomach fullness and capacity to eat decreased significantly in the eTRE + ER (*p* < 0.05; *p* < 0.001) and lTRE + ER groups (*p* < 0.05; *p* < 0.01), while desire to eat significantly decreased in the eTRE + ER (*p* < 0.05) and ER groups (*p* < 0.05). On the other hand, ghrelin levels did not change significantly during the 3INT in any group.

During the 6FU, leptin levels increased significantly in the eTRE + ER (*p* = 0.001) and lTRE + ER (*p* = 0.01) groups. However, no statistically significant differences were found between the groups. Ghrelin levels increased significantly in all three groups during the 6FU (eTRE + ER, *p* < 0.01; lTRE + ER, *p* < 0.05; ER, *p* < 0.01) with no significant differences between the groups. Moreover, fullness significantly decreased only in the lTRE + ER group (*p* < 0.01). Conversely, desire to eat and capacity to eat significantly increased in the eTRE + ER (*p* < 0.01) and ER groups (*p* < 0.05), while stomach fullness statistically increased only in the eTRE + ER group (*p* < 0.05) (Table 4).

### 3.5. Barriers to Adherence in eTRE + ER, lTRE + ER and ER in 6FU

Barriers to adherence to the dietary strategy were reported by 24 of 26 participants in the eTRE + ER group, 18 of 22 participants in the lTRE + ER group and 18 of 21 participants in the ER group. In total, participants in the eTRE + ER group reported 45 barriers, those in the lTRE + ER group reported 25, and those in the ER group 29. Detailed statements from participants are shown in Table 5.

**Figure 3:**
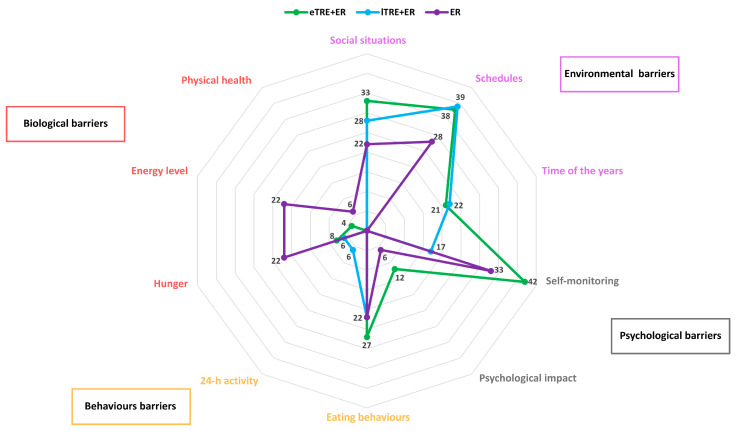
Distribution of perceived barriers to dietary adherence across biological, behavioral, psychosocial and environmental domains during the 6FU period in the eTRE + ER (n = 26), lTRE + ER (n = 22), and ER (n = 21) groups. Values represent the percentage of participants within each group reporting each barrier category. Biological barriers included hunger, low energy levels and physical health problems; behavioral barriers included changes in 24 h activity patterns and eating behaviors; psychosocial barriers included self-monitoring and psychological impact; environmental barriers included social situations, schedules and seasonal influences. eTRE—early time-restricted eating, ER—energy restriction; lTRE—late time-restricted eating.

At the biological level, three main barriers emerged: hunger, reduced energy levels and physical health problems (Figure 3). The highest proportion of reports of hunger as a barrier was in the ER group (22%), followed by the eTRE + ER group (8%) and the lTRE + ER group (6%). Reports of reduced energy levels—such as “poor well-being and fatigue”—were common in the ER group (22%), less common in the eTRE + ER group (4%) and absent in the lTRE + ER group (0%). Unexpected physical health problems were reported only by two participants in the ER group.

Psychosocial barriers were particularly pronounced in the eTRE + ER and ER groups (Figure 3). Difficulties with self-monitoring (e.g., lack of motivation) were most frequently reported in the eTRE + ER group (42%), followed by the ER group (33%), and to a lesser extent in the lTRE + ER group (17%). Psychosocial factors, including emotional eating and unexpected adverse life events, were also reported more frequently in the eTRE + ER group (12%) than in the ER group (6%), while no such barriers were reported in the lTRE + ER group (0%).

Regarding behavioral barriers, participants in the eTRE + ER group more frequently mentioned the difficulty of combining the eating window and ER, as well as having too few meals. These barriers were mentioned by 27% in the eTRE + ER group and 22% in both the lTRE + ER and ER groups. In addition, 6% of the reports in the lTRE + ER group related to changes in 24 h behavioral patterns, particularly increased physical activity.

Environmental factors were the most frequently reported category of barriers in all groups. The most commonly reported challenges included disruptions to daily routines, such as changes in work schedules, holidays, business trips and coordinating daily activities. These problems were most common in the eTRE + ER group (39%), closely followed by the lTRE + ER group (38%) and the ER group (28%). Social situations were particularly pronounced in the eTRE + ER group with 33% of reports. This was followed by the lTRE + ER group, while the ER group reported a lower proportion (22%). In addition, both TRE + ER groups (eTRE + ER: 21%, lTRE + ER: 22%) cited summertime as an environmental challenge, with the eTRE + ER group primarily citing longer daylight hours and the lTRE + ER group citing hot weather as the reason.

### 3.6. Predictors of Changes in Adherence (Eating Window and Energy Intake) and Body Mass During a 6FU

In subsequent analyses, we examined whether reported barriers predicted the changes in body mass, eating window and energy intake during the 6FU. Linear regression analyses were first conducted in the pooled sample including all three groups and then performed separately within each intervention group (eTRE + ER, lTRE + ER and ER). The results of the linear regression models for the pooled sample including all three groups are presented in Table 6. In the pooled sample, the model with biological, behavioral, psychosocial and environmental barriers as predictors significantly explained the variability in change in body mass (F = 3.781, *p* = 0.008). The model explained 14% of the variance in changes in body mass, representing a moderate effect size (R^2^ = 0.191, adjusted R^2^ = 0.141). Among individual barriers, psychosocial (β = 0.345, *p* = 0.003) and environmental barriers (β = 0.275, *p* = 0.021) emerged as statistically significant direct predictors of change in body mass.

The regression model examining barriers as predictors of change in eating window was statistically significant (F = 3.041, *p* = 0.023), explaining 11% of the variance in change in eating window, representing a small effect size (R^2^ = 0.160, adjusted R^2^ = 0.107). Behavioral barriers (β = 0.247, *p* = 0.040) and environmental barriers (β = 0.244, *p* = 0.044) were identified as statistically significant predictors of change in eating window. The model including barriers as predictors of change in energy intake was not statistically significant (R^2^ = 0.160, adjusted R^2^ = 0.107, F = 3.041, *p* = 0.931). Similarly, neither the model examining change in eating window as a predictor of change in body mass (*p* = 0.073) nor the model examining change in energy intake as a predictor of change in body mass reached statistical significance (*p* = 0.619) in the pooled sample.

**Figure 4:**
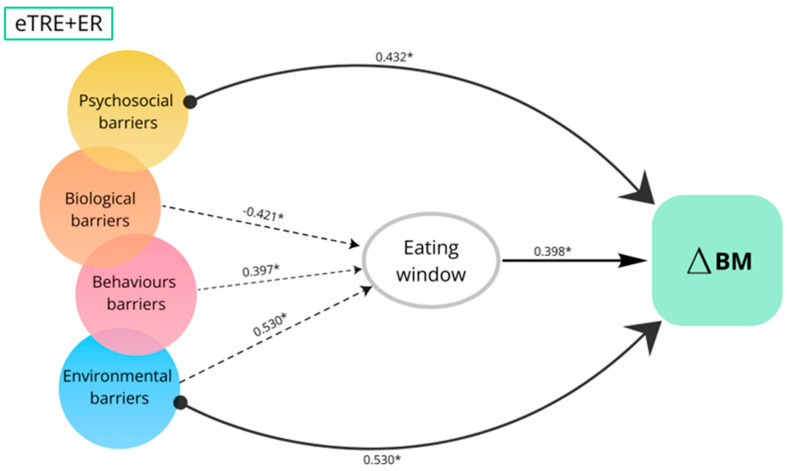
Conceptual model of barriers as direct and indirect predictors of changes in body mass (ΔBM) through changes in the eating window in the eTRE + ER group. Solid arrows represent direct associations, while dashed arrows indicate indirect associations. Values represent standardized regression coefficients (β). * Indicates statistically significant association (*p* < 0.05).

After stratifying the sample by intervention groups (eTRE + ER, lTRE + ER, and ER), statistically significant models were observed only in the eTRE + ER group, while models in the lTRE + ER and ER groups were not significant (Supplementary Tables S1–S3). The conceptual model for the eTRE + ER group is presented in Figure 4. In this group, the model including biological, behavioral, psychosocial and environmental barriers significantly explained variability in change in body mass (R^2^ = 0.402, adjusted R^2^ = 0.228, F = 3.534, *p* = 0.023).

Consistent with the findings in the pooled sample, psychosocial (β = 0.432, *p* = 0.019) and environmental barriers (β = 0.009, *p* = 0.023) also emerged as statistically significant predictors of change in body mass in the eTRE + ER group. The model including barriers as predictors of change in eating window was statistically significant and explained a large proportion of variance (R^2^ = 0.403, adjusted R^2^ = 0.289, F = 3.544, *p* = 0.023). Analyses examining predictors of change in eating window identified behavioral barriers (β = 0.397, *p* = 0.043) and environmental barriers (β = 0.540, *p* = 0.008) as significant predictors, in line with the pooled sample. In the eTRE + ER group, biological barriers (β = −0.421, *p* = 0.024) also reached statistical significance; however, this result should be interpreted cautiously due to the small number of participants reporting biological barriers (n = 2). Furthermore, the model examining change in eating window as a predictor of change in body mass explained a small proportion of variance (R^2^ = 0.158, adjusted R^2^ = 0.123, F = 4.511, *p* = 0.044). In contrast, the model including barriers as predictors of change in energy intake was not statistically significant (R^2^ = 0.161, adjusted R^2^ = 0.001, F = 1.007, *p* = 0.426). Similarly, the model examining change in energy intake as a predictor of change in body mass was not statistically significant (R^2^ = 0.066, adjusted R^2^ = 0.028, F = 1.708, *p* = 0.204), likely reflecting the relatively stable energy intake observed over the 6FU period.

As a final step, binary logistic regression models were used in the pooled sample, which include all three groups, to identify predictors of body mass maintenance during the 6FU period (Table 7). The overall model explained approximately 25.6% of the variance in the outcome (Nagelkerke R^2^ = 0.256); however, the model as a whole did not reach statistical significance (*p* = 0.120). Among the predictors included, psychosocial barriers were a statistically significant predictor of body mass maintenance (OR = 4.75, 95% CI: 1.40–16.14, *p* = 0.013).

## 4. Discussion

The primary aim of the study was to investigate whether participants of the 3INT study continued to adhere to the assigned dietary regimen (eTRE + ER, lTRE + ER, and ER) during the 6FU in a free-living condition and to identify the barriers to adherence for each dietary regimen. In addition, we aimed to determine whether changes observed during the 3INT, including changes in body mass, body composition, cardiometabolic risk factors, metabolic hormones and subjective appetite, were maintained during the 6FU.

Of the 69 participants who attended the 6FU, participants in the eTRE + ER group showed significantly greater improvements in BMI, fat mass, diastolic blood pressure and leptin concentration compared to the ER group at the completion of the 3INT and significantly greater reductions in fat mass compared to the lTRE + ER group, consistent with the results of our parent study [16]. Importantly, body mass reductions during the 3INT, primarily driven by decreases in fat mass, exceeded the ≥5% threshold considered clinically relevant for improving cardiometabolic risk [24]. The eTRE + ER group also demonstrated a marked reduction in diastolic blood pressure, which may be clinically relevant given that the reductions of approximately 10 mmHg are associated with substantial reductions in cardio-vascular risk [25]. Cardiometabolic risk factors slightly improved during the 3INT, with greater improvement in participants in the eTRE + ER group. However, it is important to note that most participants in our study had cardiometabolic markers within the normal range at baseline, which may have limited the potential for detecting further metabolic improvements during the 3INT.

During 6FU, body mass partially increased, but nonetheless, reductions from baseline remained clinically meaningful (>5%) in the eTRE + ER and lTRE + ER groups, whereas the ER group no longer met this threshold. In contrast, the clinically meaningful reduction in diastolic blood pressure observed during the intervention was not maintained during 6FU. Although between-group differences were no longer statistically significant at the 6FU, metabolic outcomes did not fully return to baseline, suggesting a partial maintenance of the intervention effects.

Despite the growing number of intervention studies on TRE and TRE + ER in the last decade, relatively few have evaluated whether the observed benefits were sustained once participants returned to free-living conditions without structured supervision [3,26,27,28]. In a previous study of 10–11 h eating window implementation in healthy participants with the help of an app for 16 weeks, benefits of weight loss, improved sleep, and subjective sense of more energy were maintained for 36 weeks after the end of the intervention [27]. Conversely, in a previous randomized study, individuals with overweight or obesity at high risk of type 2 diabetes who completed 3-month TRE showed a greater decrease in body weight than the control group, but this was not maintained after the 3-month follow-up [26]. Similarly, despite initial larger reductions in fat mass and visceral fat area after 7 weeks of the TRE compared with control group, no significant between-group differences were observed after 2 years of follow up in women with overweight/obesity [28]. In our study, the partial reversal of favorable changes in body composition, cardiometabolic risk factors, metabolic hormones, and subjective appetite at 6FU coincided with a marked decline in adherence under free-living conditions. While adherence during the 3INT was high (86%) [16], it dropped substantially, and only 11.8% of participants met the predefined adherence criterion of ≥5 days per week during the free-living 6FU. Lower long-term adherence in our study may partly be explained by the fact that participants were required to follow a combined dietary strategy (TRE + ER), which may be more demanding than TRE alone. Indeed, continuation rates during follow up reported in previous studies examining TRE without ER appeared somewhat higher [26,28,29,30]. Taken together, these results indicate that high adherence is feasible under controlled conditions, whereas maintaining adherence during free-living follow-up remains challenging. This is consistent with previous evidence showing that although structured lifestyle interventions can achieve good adherence in research settings [11,13,15,31], sustaining behavioral changes in real-world environments is considerably more difficult [32]. Given that the long-term feasibility of TRE remains insufficiently understood, research should focus on identifying the barriers that participants encounter when implementing this strategy in daily life and on developing targeted, more flexible strategies to support long-term adherence to the protocol.

Building on these observations, we aimed to better understand the factors contributing to the observed decline in adherence under real-world conditions by examining the role of perceived barriers to adherence. The observation that environmental and behavioral barriers emerged as significant predictors of eating window extension in the pooled sample indicates that long-term maintenance of dietary strategies is not determined by a single barrier but rather by the interacting effects of multiple barriers [33]. Beyond their influence on eating window duration, environmental and psychosocial barriers were also associated with less favorable changes in body mass in the pooled sample. Importantly, when analyses were stratified by dietary strategy, these associations were important primarily in the eTRE + ER group. Specifically, environmental and behavioral barriers predicted a mean extension of the eating window of approximately two hours, reflecting limited compatibility between eTRE and lifestyle patterns (such as family dinner), seasonal reasons and social situations. Indeed, it has been shown that social relationships and activities often made it difficult to perform eTRE, since most social events take place at night [34,35], and especially in the summer time, which was shown in our case. To accommodate social events, participants often shifted their eating window to later hours on weekends, despite the more favorable metabolic effects associated with eTRE [29,36]. Although eTRE aligned with circadian rhythms demonstrated short-term metabolic advantages [37,38,39], its long-term feasibility in everyday life appears limited. Notably, in the eTRE + ER group, extension of the eating window was correlated with increases in body mass among 6FU, underscoring the importance of meal timing as a determinant of long-term body mass regulation [40]. In addition, behavioral barriers were largely related to the perceived difficulty in adhering to both eTRE and ER, resulting in participants discontinuing the temporal restriction more frequently than the ER. Psychosocial barriers in the eTRE + ER group were also significant predictors of body mass change, pointing to a decline in motivation following completion of the structured intervention. Family support was found to be an important factor in maintaining motivation; however, our participants were predominantly women, and for them, changing eating practices to comply with the prescribed strategy required a coordination and adaptation to other practices such as work, child care, and other family members [41]. In addition to behavioral and psychosocial mechanisms, hormonal adaptations may have contributed to the observed difficulties in maintaining eTRE over the longer term. In this group, leptin levels were reduced more markedly throughout the study compared with the other two groups. Although reductions in leptin are associated with weight loss, evidence on leptin sensitivity suggests that moderate rather than pronounced decreases may be more effective for long-term obesity management [42,43,44]. Furthermore, alterations in appetite-regulating hormones, particularly elevated ghrelin, are associated with increased subjective appetite in all three groups [42,45].

In contrast, participants in the lTRE + ER group reported the fewest perceived barriers, which may help explain the smaller extension of the eating window and relatively better adherence observed during 6FU. Notably, the lTRE + ER group was the only group that did not demonstrate statistically significant within-group increases in body mass, BMI, fat mass, waist circumference, blood pressure, fasting glucose, LDL cholesterol, or triacylglycerol’s during 6FU. Consistent with this interpretation, previous studies suggest that lTRE + ER may be more feasible than eTRE + ER, as it aligns more closely with family routines and social eating patterns [5,46].

Participants in the ER group most frequently reported biological barriers, including low energy levels, reduced well-being, and increased hunger, which they attributed to ER and an insufficient number of daily meals. Despite being commonly reported, these barriers did not emerge as statistically significant predictors of changes in body mass, eating window, or energy intake. A potential explanation for the increase in hunger in ER group may be the significant reduction in protein intake observed during the 6FU. Results of a previous meta-analysis show that higher protein intake suppresses appetite, decreases ghrelin levels, and increases levels of cholecystokinin (CCK) and glucagon-like peptide-1 (GLP-1). CCK and GLP-1 are gut-derived hormones that promote satiety by slowing gastric emptying and signaling the brain to reduce food intake, thereby contributing to appetite regulation and energy balance [44]. All the above enlisted factors likely contributed to the slightly increased energy intake observed during the follow-up.

Overall, the long-term success of TRE + ER, similar to other dietary interventions, depends on an individual’s ability to sustain adherence to the prescribed dietary regimen, which is often challenging in everyday life. A discrepancy between the prescribed eating window (eTRE or lTRE) and daily routines or social eating events decreases its practicality [47]. Previous studies have also suggested that the choice between eTRE and lTRE should be guided by individual circumstances to enhance long-term sustainability [43]. Accordingly, in our 3INT, participants were allocated to dietary regimens based on chronotype to better align the eating window with their lifestyle and thereby promote adherence. Despite this individualized allocation, follow-up results demonstrated low adherence, indicating that chronotype-based adjustment of the eating window is insufficient to maintain TRE under free-living conditions. Consistent with our findings, another study reported that a self-selected eating window does not necessarily resolve conflicts between TRE and social situations [46].

In conclusion, during the free-living 6FU, differences between dietary strategies gradually diminished, although some remained still clinically meaningful. Long-term adherence was limited across all three dietary strategies, with psychosocial, environmental, and behavioral barriers particularly evident in the eTRE + ER group and associated with a mean extension of the eating window of approximately two hours. Overall, these findings underscore the importance of accounting for long-term feasibility when implementing dietary interventions and suggest that sustained professional behavioral support may facilitate the preservation of metabolic benefits over time. However, additional research is required to confirm long-term feasibility and adherence before TRE + ER interventions can be effectively implemented into clinical practice.

Some limitations of our study should be acknowledged. First, reasons for non-attendance at the 6FU assessments could not be obtained. Therefore, the possibility of selection bias cannot be excluded, as participants who returned for 6FU may have differed systematically from those lost to 6FU. Nonetheless, the 3INT outcomes of the three groups based on prescribed intervention in 3INT were comparable to the outcomes of the groups of all participants that concluded the intervention. Moreover, attrition over time resulted in a reduced sample size at 6FU, which may have limited statistical power and the ability to detect between-group differences. A post hoc power analysis for the follow-up sample (n = 69) indicated an achieved statistical power of 0.578, suggesting limited power to detect between-group effects. Therefore, the absence of statistically significant effects should be interpreted with caution. Second, dietary intake was only recorded for 3 days during the 6FU, which is relatively low for a detailed analysis of energy intake and macronutrients. Moreover, as with all self-reported dietary assessment methods, these data are subject to measurement error and under- or over-reporting, particularly under free-living conditions. Adherence to the dietary strategies and subjective appetite were assessed using self-reported measures. Consequently, changes in reported energy intake, macronutrient composition, adherence, and appetite may not fully reflect actual eating behaviors and should be interpreted with caution. Third, the study sample was predominantly female (80%), which may limit the generalizability of the findings. Fourth, the 6FU phase was observational in nature, as participants returned to free-living conditions after completion of the intervention, which limits causal inference regarding adherence and metabolic outcomes during this period. Lastly, metabolic hormone levels and subjective appetite were measured only in the fasting state, without assessments before and after meals. Given that hormonal responses and appetite regulation are influenced by food intake, future studies incorporating multiple assessments across the day may provide a more comprehensive understanding of the physiological mechanisms underlying long-term adherence and metabolic changes.

## Figures and Tables

**Table 1:** Baseline characteristics of the participants that responded to the 6FU invitation.

	All Participants (n = 69)	eTRE + ER (n = 26)	lTRE + ER (n = 22)	ER (n = 21)	*p*-Value
**Socio-demographic determinants**					
**Sex**					0.531
Female	48 (69.6%)	20 (76.9%)	15 (68.2%)	13 (61.9%)	
Male	21 (30.4%)	6 (23.1%)	7 (31.8%)	8 (38.1%)	
**Age** (years)	46 ± 9	44 ± 9	47 ± 11	48 ± 7	0.201
**Education**					0.618
Secondary school	15 (21.7%)	4 (15.4%)	4 (18.2%)	7 (33.3%)	
Bachelor	37 (53.6%)	13 (46.2%)	13 (59.1%)	11 (52.4%)	
Master’s degree	15 (21.7%)	8 (30.8%)	4 (18.2%)	8 (38.1%)	
Doctorate degree	2 (2.9%)	1 (3.8%)	1 (4.5%)	3 (14.3%)	
**Place of residence**					**0.026** **^a^**
Village	17 (24.6%)	12 (46.2%)	2 (9.1%)	3 (14.3%)	
Suburban area	17 (24.6%)	4 (15.4%)	6 (27.3%)	7 (33.3%)	
City	35 (50.7%)	10 (38.5%)	14 (63.6%)	11 (52.4%)	
**Working status**					0.471
Employed	66 (95.6%)	25 (96.2%)	20 (90.9%)	21 (100%)	
Unemployed	1 (1.4%)	0 (0%)	1 (4.5%)	0 (0%)	
Student	2 (2.9%)	1 (3.8%)	1 (4.5%)	0 (0%)	
**Body composition**					
Body mass (kg)	85.8 ± 12.4	85.3 ± 11.7	86.8 ± 9.6	85.1 ± 15.7	0.877
BMI (kg/m^2^)	29.0 ± 3.0	28.9 ± 3.0	29.3 ± 2.2	28.9 ± 3.8	0.880
Waist circumference (cm)	96.4 ± 10.6	94.6 ± 9.4	98.6 ± 7.6	96.4 ± 14.2	0.423
Fat mass (kg)	28.4 ± 6.9	28.7 ± 6.4	29.0 ± 5.4	27.5 ± 8.9	0.773
Fat-free mass (kg)	57.3 ± 10.6	56.7 ± 10.7	57.9 ± 10.3	57.5 ± 11.2	0.916
Muscle mass (kg)	54.4 ± 10.1	53.8 ± 10.2	55.0 ± 10.3	54.6 ± 10.6	0.916
**Blood pressure**					
Systolic blood pressure (mmHg)	130 ± 14	135 ± 14	130 ± 12	125 ± 14	0.056
Diastolic blood pressure (mmHg)	85 ± 9	88 ± 8	84 ± 9	81 ± 10	**0.029** **^b^**
**Cardiometabolic risk factors**					
Fasting glucose (mmol/L)	5.24 ± 0.62	5.17 ± 0.58	5.35 ± 0.43	5.20 ± 0.83	0.597
Total cholesterol (mmol/L)	5.37 ± 1.05	5.07 ± 1.02	5.87 ± 1.20	5.25 ± 0.75	**0.025** **^a^**
HDL cholesterol (mmol/L)	1.58 ± 0.41	1.58 ± 0.47	1.56 ± 0.38	1.60 ± 0.40	0.948
LDL cholesterol (mmol/L)	3.92 ± 1.09	3.59 ± 0.91	4.40 ± 1.40	3.85 ± 0.76	**0.033** **^a^**
Triacylglycerol (mmol/L)	1.46 ± 1.41	1.36 ± 1.62	1.77 ± 1.71	1.26 ± 0.51	0.458
**Metabolic hormones**					
Leptin (ng/mL)	80.8 ± 52.4	98.4 ± 60.6	77.0 ± 45.7	63.8 ± 43.6	0.084
Ghrelin (pg/mL)	21.2 ± 24.4	27.1 ± 26.5	15.1 ±15.2	20.4 ± 29.2	0.274
**Resting metabolic rate (kcal)**	1610 ± 347	1612 ± 363	1654 ± 320	1560 ± 364	0.680
**Food intake**					
Daily energy intake (kcal per day)	2217 ± 587	2290 ± 773	2108 ± 510	2239 ± 314	0.559
Fats (%)	37.2 ± 5.4	36.2 ± 5.8	37.8 ± 4.0	38.0 ± 6.1	0.478
Protein (%)	16.3 ± 2.9	16.0 ± 2.4	16.8 ± 3.1	16.2 ± 3.2	0.653
Carbohydrates (%)	44.5 ± 6.1	45.9 ± 6.3	43.0 ± 4.7	44.2 ± 6.8	0.237
**Subjective appetite (1–10)**					
Hunger	5.6 ± 1.6	5.7 ± 2.0	5.7 ± 1.2	5.5 ± 1.6	0.921
Fullness	5.8 ± 1.7	5.7 ± 2.0	6.0 ± 1.4	5.6 ± 1.7	0.745
Stomach fullness	4.4 ± 2.5	4.6 ± 2.4	4.9 ± 2.3	3.6 ± 2.8	0.203
Desire to eat	5.3 ± 2.3	5.6 ± 2.1	5.0 ± 2.5	5.0 ± 2.4	0.702
Capacity to eat	5.2 ± 2.3	5.8 ± 2.3	4.8 ± 2.4	4.9 ± 2.2	0.248
**Eating window** (h)	13 ± 1	12 ± 1	13 ± 1	13 ± 1	0.119

Note: BMI = body mass index; eTRE = early time-restricted eating, ER = energy restriction; lTRE = late time-restricted eating; n = number. Data are present as N (%) or means ± SD. The *p* value indicates a significant (*p* < 0.05) difference between the eTRE + ER, lTRE + ER and ER groups, using ANOVA for normally distributed data, the Kruskal–Wallis test for non-normally distributed data, and the chi-square test for categorical variables. *p* ^a^—indicates a significant (*p* < 0.05) difference between the eTRE + ER and lTRE + ER groups. *p* ^b^—indicates a significant (*p* < 0.05) difference between the eTRE + ER and ER groups.

**Table 2:** Changes in energy intake, macronutrient distribution, and eating window from baseline to the 3INT, and from end of the 3INT to the 6FU.

Variables	eTRE + ER (n = 26)	lTRE + ER (n = 22)	ER (n = 21)	*p*-Value
^1^ Energy intake 3INT change (kcal) ^2^ Energy intake 6FU change (kcal)	−660 ± 470 *** 70 ± 320	−510 ± 630 ** 90 ± 300	−630 ± 310 *** 270 ± 370 ^##^	0.553 0.102
^1^ Carbohydrates intake 3IN change (%) ^2^ Carbohydrates intake 6FU change (%)	−1.0 ± 7.1 −0.6 ± 6.3	−0.8 ± 9.4 −0.9 ± 8.1	−0.9 ± 7.2 1.9 ± 5.9	0.995 0.344
^1^ Protein intake 3IN change (%) ^2^ Protein intake 6FU change (%)	4.2 ± 3.2 *** −1.0 ± 4.4	3.5 ± 3.8 *** −2.8 ± 3.8 ^##^	4.9 ± 3.8 *** −4.4 ± 3.1 ^###^	0.411 **0.014 ^b^**
^1^ Fats intake 3IN change (%) ^2^ Fats intake 6FU change (%)	−2.4 ± 6.8 0.3 ± 7.5	−2.2 ± 9.5 3.0 ± 8.1	−3.9 ± 7.3 * 1.8 ± 5.1	0.751 0.408
^1^ Eating window 3IN change (h) ^2^ Eating window 6FU change (h)	−4.8 ± 1.0 *** 2.0 ± 1.8 ^###^	−4.3 ± 1.3 *** 0.7 ± 1.0 ^##^	−1.0 ± 1.2 ** 0.0 ± 1.0	**<0.001 ^b,c^** **<0.001 ^a,b^**
^1^ Resting metabolic rate 3IN change (kcal) ^2^ Resting metabolic rate 6FU change (kcal)	−200 ± 280 ** 14 ± 280	−110 ± 200 * −57 ± 220	−90 ± 200 33 ± 240	0.219 0.521

Note: eTRE = early time-restricted eating; ER = energy restriction; lTRE = late time-restricted eating; 3INT = 3-month intervention; 6FU = 6-month follow up. Changes are shown as means ± SD. ^1^ Changes in parameters during interventions were calculated as the difference between values at the end of the 3INT and baseline values. ^2^ Here, 6FU changes were calculated as the differences in each parameter between the 6FU and the end of the 3INT. * Significant differences within each group from baseline to the 3INT (paired t-test or Wilcoxon test): *** *p* < 0.001, ** *p* < 0.01, * *p* < 0.05. ^#^ Significant differences within each group from the 3INT to the 6FU (paired t-test or Wilcoxon test): ^###^ *p* < 0.001, ^##^ *p* < 0.01. Bold values indicate statistically significant differences between groups (one-way ANOVA with post hoc analysis or Kruskal–Wallis’s test). *p* ^a^—denotes significant (*p* < 0.05) difference between eTRE + ER and lTRE + ER groups. *p* ^b^—denotes significant (*p* < 0.05) difference between eTRE + ER and ER group. *p* ^c^—denotes significant (*p* < 0.05) difference between lTRE + ER and ER group.

**Table 3:** Changes In body mass, body composition, and cardiometabolic risk factors from baseline to the 3INT and from the end of 3INT to 6FU.

Variables	eTRE + ER (n = 26)	lTRE + ER (n = 22)	ER (n = 21)	*p*-Value
**Body composition**				
^1^ Body mass 3INT change (kg) ^2^ Body mass 6FU change (kg)	−7.3 ± 2.6 *** 2.1 ± 2.9 ^##^	−6.2 ± 2.5 *** 0.9 ± 2.5	−5.5 ± 3.1 *** 1.8 ± 2.2 ^##^	0.064 0.264
^1^ BMI 3INT change (kg/m^2^) ^2^ BMI 6FU change (kg/m^2^)	−2.5 ± 0.9 *** 0.7 ± 1.0 ^###^	−2.1 ± 0.8 *** 0.3 ± 0.8	−1.8 ± 1.1 *** 0.6 ± 0.7 ^##^	**0.036 ^b^** 0.236
^1^ Fat mass 3INT change (kg) ^2^ Fat mass 6FU change (kg)	−5.8 ± 2.5 *** 1.6 ± 2.5 ^##^	−4.0 ± 1.9 *** 0.8 ± 2.0	−4.0 ± 2.5 *** 1.6 ± 1.7 ^###^	**0.013 ^a,b^** 0.224
^1^ Waist circumference 3INT change (cm) ^2^ Waist circumference 6FU change (cm)	−5.5 ± 2.7 *** 0.7 ± 1.0	−5.3 ± 3.0 *** -0.4 ± 2.3	−4.1 ± 2.0 *** 1.1 ± 1.5 ^##^	0.143 0.064
^1^ Fat-free mass 3INT change (kg) ^2^ Fat-free mass 6FU change (kg)	−1.6 ± 1.2 *** 0.5 ± 1.3	−2.2 ± 1.2 *** 0.1 ± 1.0	−1.5 ± 1.5 *** 0.2 ± 1.2	0.173 0.503
^1^ Muscle mass 3INT change (kg) ^2^ Muscle mass 6FU change (kg)	−1.6 ± 1.1 *** 0.5 ± 1.3	−2.1 ± 1.2 *** 0.1 ± 0.9	−1.4 ± 1.4 *** 0.1 ± 1.2	0.176 0.471
**Blood pressure**				
^1^ Systolic blood pressure 3INT change (mmHg) ^2^ Systolic blood pressure 6FU change (mmHg)	−11 ± 11 *** 5 ± 12 ^#^	−10 ± 14 ** 5 ± 12	−5 ± 12 5 ± 10 ^#^	0.289 0.999
^1^ Diastolic blood pressure 3INT change (mmHg) ^2^ Diastolic blood pressure 6FU change (mmHg)	−12 ± 8 *** 5 ± 8 ^##^	−9 ± 9 *** 4 ± 8	−6 ± 7 *** 1 ± 8	**0.037 ^b^** 0.281
**Cardiometabolic risk factors**				
^1^ Fasting glucose 3INT change (mmol/L) ^2^ Fasting glucose 6FU change (mmol/L)	−0.42 ± 0.60 ** 0.19 ± 0.41 ^#^	−0.09 ± 0.25 0.00 ± 0.34	−0.29 ± 0.59 * 0.22 ± 0.59	0.091 0.276
^1^ Total cholesterol 3INT change (mmol/L) ^2^ Total cholesterol 6FU change (mmol/L)	−0.31 ± 0.55 ** 0.24 ± 0.55 ^#^	−0.11 ± 0.60 −0.01 ± 0.72	−0.22 ± 0.58 −0.09 ± 0.72	0.513 0.169
^1^ LDL cholesterol 3INT change (mmol/L) ^2^ LDL cholesterol 6FU change (mmol/L)	−0.24 ± 0.33 *** 0.24 ± 0.44 ^#^	−0.03 ± 0.72 0.02 ± 0.68	−0.30 ± 0.71 0.04 ± 0.68	0.311 0.363
^1^ HDL cholesterol 3INT change (mmol/L) ^2^ HDL cholesterol 6FU change (mmol/L)	−0.06 ± 0.24 0.42 ± 0.21 ^###^	−0.07 ± 0.04 * 0.41 ± 0.25 ^###^	−0.08 ± 0.20 0.38 ± 0.26 ^###^	0.952 0.842
^1^ Triacylglycerol 3INT change (mmol/L) ^2^ Triacylglycerol 6FU change (mmol/L)	−0.32 ± 0.90 * 0.10 ± 0.38	−0.28 ± 0.56 * −0.14 ± 0.61	−0.07 ± 0.34 −0.02 ± 0.42	0.405 0.223

Note: BMI = body mass index; HDL = high-density lipoprotein; LDL = low-density lipoprotein; eTRE = early time-restricted eating; ER = energy restriction; lTRE = late time-restricted eating; 3INT = 3-month intervention; 6FU = 6-month follow up. Changes are shown as means ± SD. ^1^ Changes in parameters during interventions were calculated as the difference between values at the end of the 3INT and baseline values. ^2^ Follow-up changes were calculated as the difference in each parameter between the 6FU and the end of the 3INT. * Significant differences within each group from baseline to the 3INT (paired t-test or Wilcoxon test): *** *p* < 0.001, ** *p* < 0.01, * *p* < 0.05. ^#^ Significant differences within each group from the 3INT to the 6FU (paired t-test or Wilcoxon test): ^###^ *p* < 0.001, ^##^ *p* < 0.01, ^#^ *p* < 0.05. Bold values indicate statistically significant differences between groups (one-way ANOVA with post hoc analysis or Kruskal–Wallis’s test). *p* ^a^—denotes a significant (*p* < 0.05) difference between eTRE + ER and lTRE + ER groups. *p* ^b^—denotes a significant (*p* < 0.05) difference between eTRE + ER and ER groups.

**Table 4:** Changes in metabolic hormones and subjective appetite from baseline to the 3INT and from the end of 3INT to the 6FU.

Variables	eTRE + ER	lTRE + ER	ER	*p*-Value
**Metabolic hormones**				
^1^ Leptin 3INT change (ng/mL) ^2^ Leptin 6FU change (ng/mL)	−53.3 ± 36.4 *** 24.9 ± 26.2 ^###^	−31.2 ± 18.8 *** 14.4 ± 17.8 ^##^	−22.7 ± 23.4 *** 10.5 ± 25.0	**0.002 ^a^** 0.111
^1^ Ghrelin 3INT change (pg/mL) ^2^ Ghrelin 6FU change (pg/mL)	2.4 ± 13.8 18.5 ± 31.2 ^##^	11.2 ± 26.6 10.4 ± 20.1 ^#^	5.3 ± 16.1 21.8 ± 30.1 ^##^	0.336 0.427
**Subjective appetite (1–10)**				
^1^ Hunger 3INT change ^2^ Hunger 6FU change	−1.5 ± 2.7 ** 0.6 ± 2.8	−2.0 ± 2.6 ** 0.7 ± 2.5	−1.7 ± 2.3 ** 0.4 ± 1.9	0.825 0.924
^1^ Fullness 3INT change ^2^ Fullness 6FU change	0.9 ± 2.2 −0.8 ± 2.4	1.0 ± 1.9 * −1.5 ± 2.0 ^##^	0.7 ± 2.3 −0.3 ± 2.0	0.890 0.184
^1^ Stomach fullness 3INT change ^2^ Stomach fullness 6FU change	−1.7 ± 2.3 ** 1.3 ± 2.3 ^#^	−1.1 ± 2.3 * 0.2 ± 2.4	−0.1 ± 1.7 0.5 ± 2.3	0.054 0.232
^1^ Desire to eat 3INT change ^2^ Desire to eat 6FU change	−1.5 ± 2.0 ** 1.2 ± 2.8 ^#^	−1.1 ± 3.1 0.7 ± 2.0	−1.4 ± 2.1 ** 1.4 ± 2.7 ^#^	0.869 0.672
^1^ Capacity to eat 3INT change ^2^ Capacity to eat 6FU change	−2.0 ± 3.0 ** 1.7 ± 2.8 ^##^	−1.3 ± 2.3 * 0.8 ± 2.0	−0.8 ± 2.3 0.8 ± 1.7 ^#^	0.267 0.250

Note: BMI = body mass index; HDL = high-density lipoprotein; LDL = low-density lipoprotein; eTRE = early time-restricted eating; ER = energy restriction; lTRE = late time-restricted eating; 3INT = 3-month intervention; 6FU = 6-month follow up. Changes are shown as means ± SD. ^1^ Changes in parameters during interventions were calculated as the difference between values at the end of the 3INT and baseline values. ^2^ Follow-up changes were calculated as the difference in each parameter between the 6FU and the end of the 3INT. * Significant differences within each group from baseline to the 3INT (paired t-test or Wilcoxon test): *** *p* < 0.001, ** *p* < 0.01, * *p* < 0.05. ^#^ Significant differences within each group from the 3INT to the 6FU (paired t-test or Wilcoxon test): ^###^ *p* < 0.001, ^##^ *p* < 0.01, ^#^ *p* < 0.05. Bold values indicate statistically significant differences between groups (one-way ANOVA with post hoc analysis or Kruskal–Wallis’s test). *p* ^a^—denotes a significant (*p* < 0.05) difference between eTRE + ER and ER groups.

**Table 5:** Barriers to adherence to eTRE + ER, lTRE + ER and ER in 6FU.

		eTRE + ER	lTRE + ER	ER
**Environmental** **barriers**	Social situations	“My social life makes it difficult for me to follow TRE”. “Cooking for the whole family, where dinners are served later than 4 p.m.” “My children prefer a different eating schedule.”	“Social life.”	“Way of life (social life, family).” “I’ve heard opinions that I’m too skinny.”
	Schedules	“Balancing work and university commitments.” “A different schedule in the new job.” “Business trips.” “A several-week vacation has changed my eating routine.” “Difficulty in balancing work schedule, meal preparation, and the time window for TRE.”	“Work—fieldwork.” “Two jobs—the concept of coming home, with hours not aligning with TRE.” “Vacation and with it, a changed rhythm and schedule of life.” “Business trips.”	“Aligning diet with work schedule.” “Vacation—changed rhythm.”
	Time of year	“Too long days during summer for TRE.” “Hot weather.” “Due to the season, she has eased up on her energy restriction.”	“Hot weather.” “More ice cream and cold desserts due to the summer.”	
**Psychosocial** **barriers**	Self-monitoring	“Lack of motivation and a drop in enthusiasm.”	“After the vacation, I had an excuse every week to postpone the diet.” “Lack of motivation.”	“Lack of motivation.” “Laziness in counting calories.”
	Psychological impact	“Unexpected unpleasant events in life.”		“Emotional eating.”
**Behaviors** **barriers**	Eating behaviors	“It’s too demanding to follow both TRE and ER.” “Excessive energy restriction.” “Too few meals.”	“Lack of persistence in counting calories.” “Too short of an eating window (I would like to have a longer eating period than 8 h).” “Inadequate eating window.” “Too demanding to follow both ER and TRE.”	“Too few meals (3 meals).” “The intention is always good, I plan my meals and prepare healthy dishes, but by the evening, it often falls apart.”
	24 h activity behaviors		“Increased physical activity.”	
**Biological** **barriers**	Hunger	“Hunger.”	“Hunger.”	“I no longer stick to ER because I’m hungry.”
	Energy	“Poor well-being and fatigue.”		“Poor well-being and fatigue.”
	Physical health			“Changes in health status.”

**Table 6:** Predictors of changes in body mass, eating window, and energy intake in all three groups during 6FU (n = 26).

Variables	B	SE	ß	*t*	*p*	R^2^	Adj. R^2^	F	*p*-Value
**Barriers as predictor of ∆ BM**									
Biological	0.404	0.541	0.084	0.746	0.458				
Behaviors	0.259	0.559	0.054	0.464	0.645				
Psychosocial	1.667	0.544	0.345	3.068	**0.003**	0.191	0.141	3.781	**0.008**
Environmental	0.714	0.302	0.275	2.367	**0.021**				
**Barriers as predictor of ∆ EW**									
Biological	−0.669	0.339	−0.227	−1.972	0.053				
Behaviors	0.735	0.350	0.247	2.099	**0.040**				
Psychosocial	−0.174	0.341	−0.059	−0.512	0.610	0.160	0.107	3.041	**0.023**
Environmental	0.389	0.189	0.244	2.059	**0.044**				
**Barriers as predictor of ∆ EI**									
Biological	55.908	78.750	0.089	0.710	0.480				
Behaviors	10.970	81.378	0.017	0.135	0.893				
Psychosocial	−28.345	79.087	−0.045	−0.358	0.721	0.013	−0.049	0.211	0.931
Environmental	−14.332	43.891	−0.042	−0.327	0.745				

Note: Adj. R^2^ = adjusted coefficient of determination; B = unstandardized regression coefficient; F = F-statistic; R^2^ = coefficient of determination; SE = standard error; *t* = *t*-value; ΔBM = change in body mass; ΔEW = change in eating window; ΔEI = change in energy intake; ß = Standardized regression coefficient.

**Table 7:** Predictors of body mass maintenance during the 6FU in the pooled sample.

Variables	B	SE	Wald	OR	95% Cl	*p*-Value
**Biological barriers**	0.667	0.608	1.204	1.949	0.592, 6.418	0.272
**Behavioral barriers**	1.146	0.715	2.569	3.146	0.775, 12.776	0.109
**Psychosocial barriers**	1.558	0.624	6.229	4.749	1.397, 16.139	**0.013**
**Environmental barriers**	0.586	0.338	3.003	1.798	0.926, 3.490	0.083

Note: B = regression coefficient; SE = standard error; Wald = Wald statistics; OR = odds ratio; 95% CI = confidence interval; ∆EW = change in eating window; ∆EI = change in energy intake.

## Data Availability

Data from this article will be made available upon reasonable request from the corresponding author. The data are not publicly available due to ongoing study.

## Supplementary Materials

The following supporting information can be downloaded at: https://www.mdpi.com/article/10.3390/nu18061004/s1, Table S1: Predictors of changes in body mass, eating window and energy intake in the eTRE + ER group in 6FU (n = 26); Table S2: Predictors of changes in body mass, eating window and energy intake in the lTRE + ER group in 6FU (n = 26); Table S3: Predictors of changes in body mass, eating window and energy intake in the ER group in 6FU (n = 26).

## Abbreviations

The following abbreviations are used in this manuscript:

BMI	Body mass index
ER	Energy restriction
eTRE	Early time-restricted eating
HDL	High-density lipoprotein
lTRE	Late time-restricted eating
LDL	Low-density lipoprotein
TRE	Time-restricted eating
3INT	3-month intervention
6FU	6-month follow up
