# Supplementary material for: Adherence and Metabolic Outcomes of Early and Late Time-Restricted Eating with Energy Restriction vs. Energy Restriction Alone: A 6-Month Follow-Up
Source: Nutrients. 2026 Mar 21;18(6):1004. doi: 10.3390/nu18061004 (PMC13029005; doi:10.3390/nu18061004)
Supplement: Supplementary file 1 [file nutrients-18-01004-s001.zip › nutrients-4187768-supplementary.pdf]

Table S1: Predictors of changes in body mass, eating window and energy intake in the the eTRE+ER group at 6FU (n = 26).

**Variables**	**B**	**SE**	**ß**	**t**	**p**	**R^2**	**Adj. R^2**	**F**	***p*-value**
**Barriers as predictor of Δ BM**									
Biological	-1.369	1.166	-0.203	-1.174	0.254	0.402	0.228	3.534	**0.023**
Behaviors	0.710	0.775	0.169	0.916	0.370				
Psychosocial	2.152	0.845	0.432	2.546	**0.019**				
Environmental	1.513	0.525	0.530	2.880	**0.009**				
**Barriers as predictor of Δ EW**									
Biological	-1.722	0.708	-0.421	-2.432	**0.024**	0.403	0.289	3.544	**0.023**
Behaviors	1.015	0.470	0.397	2.159	**0.043**				
Psychosocial	-0.348	0.513	-0.115	-0.679	0.505				
Environmental	0.937	0.319	0.540	2.938	**0.008**				
**Barriers as predictor of Δ EI**									
Biological	1.168	154.673	0.002	0.008	0.994	0.161	0.001	1.007	0.426
Behaviors	67.496	102.726	0.143	0.657	0.518				
Psychosocial	79.541	112.102	0.143	0.710	0.486				
Environmental	130.949	69.665	0.410	1.880	0.074				
**Δ EW as predictor of Δ BM**									
EW	0.242	0.114	0.398	2.124	**0.044**	0.158	0.123	4.511	**0.044**
**Δ EI as predictor of Δ BM**									
EI	22.246	22.071	0.258	1.307	0.204	0.066	0.028	1.708	0.204

Note: Adj. R^2 = Adjusted coefficient of determination; B = Unstandardized regression coefficient; F = F-statistic; R^2 = Coefficient of determination; SE = standard error; t = t-value; ΔBM = change in body mass; ΔEW = change in eating window; ΔEI = change in energy intake; ß = Standardized regression coefficient.

Table S2: Predictors of changes in body mass, eating window and energy intake in the lTRE+ER group at 6FU (n = 26).

Variables	B	SE	ß	t	p	R^2	Adj. R^2	F	*p*-value
**Barriers as predictor of Δ BM**									
Biological	6.622	2.399	0.570	2.761	0.013	0.333	0.176	2.118	0.123
Behaviors	-0.161	1.158	-0.028	-0.139	0.891				
Psychosocial	1.218	1.419	0.173	0.858	0.403				
Environmental	0.725	0.746	0.200	0.972	0.345				
**Barriers as predictor of Δ EW**									
Biological	2.578	0.980	0.546	2.631	0.018	0.325	0.166	2.044	0.134
Behaviors	0.575	0.473	0.245	1.215	0.241				
Psychosocial	-0.157	0.580	-0.055	-0.271	0.790				
Environmental	0.044	0.305	0.040	0.143	0.888				
**Barriers as predictor of Δ EI**									
Biological	-120.732	356.005	-0.084	-0.339	0.739	0.034	-0.193	0.150	0.960
Behaviors	5.138	171.868	0.007	0.030	0.976				
Psychosocial	-146.059	210.637	-0.168	-0.693	0.497				
Environmental	-15.926	110.669	-0.036	-0.144	0.887				
**Δ EW as predictor of Δ BM**									
EW	0.045	0.090	0.110	0.496	0.625	0.012	-0.037	0.246	0.625
**Δ EI as predictor of Δ BM**									
EI	11.128	27.472	0.090	1.405	0.690	0.008	-0.041	0.164	0.690

Note: Adj. R^2 = Adjusted coefficient of determination; B = Unstandardized regression coefficient; F = F-statistic; R^2 = Coefficient of determination; SE = standard error; t = t-value; ΔBM = change in body mass; ΔEW = change in eating window; ΔEI = change in energy intake; ß = Standardized regression coefficient.

Table S3: Predictors of changes in body mass, eating window and energy intake in the ER group at 6FU (n = 26).

Variables	B	SE	ß	t	p	R^2	Adj. R^2	F	p
**Barriers as predictor of Δ BM**									
Biological	0.179	0.676	0.062	0.265	0.794	0.171	-0.037	0.823	0.529
Behaviors	1.225	1.348	0.227	0.909	0.377				
Psychosocial	1.467	0.907	0.390	1.617	0.125				
Environmental	0.441	0.427	0.251	1.032	0.317				
**Barriers as predictor of Δ EW**									
Biological	-0.302	0.319	-0.219	-0.948	0.357	0.191	-0.011	0.946	0.463
Behaviors	0.029	0.636	0.011	0.046	0.964				
Psychosocial	-0.697	0.428	-0.388	-1.629	0.123				
Environmental	0.020	0.201	0.024	0.098	0.923				
**Barriers as predictor of Δ EI**									
Biological	-58.546	113.570	-0.119	-0.516	0.613	0.187	-0.016	0.920	0.476
Behaviors	58.367	226.435	0.064	0.258	0.800				
Psychosocial	-101.059	152.451	-0.158	-0.663	0.517				
Environmental	-115.051	71.729	-0.387	-1.604	0.128				
**Δ EW as predictor of Δ BM**									
EW	-0.099	0.107	-0.207	-0.921	0.369	0.043	-0.008	0.848	0.369
**Δ EI as predictor of Δ BM**									
EI	-45.740	37.498	-0.269	1.488	0.237	0.073	0.024	1.488	0.273

Note: Adj. R^2 = Adjusted coefficient of determination; B = Unstandardized regression coefficient; F = F-statistic; R^2 = Coefficient of determination; SE = standard error; t = t-value; ΔBM = change in body mass; ΔEW = change in eating window; ΔEI = change in energy intake; ß = Standardized regression coefficient.
